# Patient and surgical prognostic factors for inpatient functional recovery following THA and TKA: a prospective cohort study

**DOI:** 10.1186/s13018-020-01854-9

**Published:** 2020-08-27

**Authors:** Nicola A. Hewlett-Smith, Rodney P. Pope, Wayne A. Hing, Vini P. Simas, James W. Furness

**Affiliations:** 1 Faculty of Health Sciences and Medicine, Bond University, Gold Coast, Australia; 2 Nicola Hewlett-Smith Allied Health Department, The Wesley Hospital, PO Box 499, Brisbane, 4066 Australia; 3 School of Community Health, Charles Sturt University, Albury, Australia

**Keywords:** Total hip arthroplasty, Total knee arthroplasty, Prognostic factors, Predictors, Inpatient function, Functional recovery

## Abstract

**Background:**

The introduction of enhanced recovery pathways has demonstrated both patient and organisational benefits. However, enhanced recovery pathways implemented for total hip arthroplasty (THA) and total knee arthroplasty (TKA) vary between health-care organisations, as do their measures of success, particularly patient-related outcomes. Despite inpatient functional recovery being essential for safe and timely hospital discharge, there is currently no gold standard method for its assessment, and the research undertaken to establish prognostic factors is limited. This study aimed to identify prognostic factors and subsequently develop prognostic models for inpatient functional recovery following primary, unilateral THA and TKA; identify factors associated with acute length of stay; and assess the relationships between inpatient function and longer-term functional outcomes.

**Methods:**

Correlation and multiple regression analyses were used to determine prognostic factors for functional recovery (assessed using the modified Iowa Level of Assistance Scale on day 2 post-operatively) in a prospective cohort study of 354 patients following primary, unilateral THA or TKA.

**Results:**

For the overall cohort and TKA group, significant prognostic factors included age, sex, pre-operative general health, pre-operative function, and use of general anaesthesia, local infiltration analgesia, and patient-controlled analgesia. In addition, arthroplasty site was a prognostic factor for the overall cohort, and surgery duration was prognostic for the TKA group. For the THA group, significant prognostic factors included pre-operative function, Risk Assessment and Prediction Tool score, and surgical approach. Several factors were associated with acute hospital length of stay. Inpatient function was positively correlated with functional outcomes assessed at 6 months post-operatively.

**Conclusions:**

Prognostic models may facilitate the prediction of inpatient flow thus optimising organisational efficiency. Surgical prognostic factors warrant consideration as potential key elements in enhanced recovery pathways, associated with early post-operative functional recovery. Standardised measures of inpatient function serve to evaluate patient-centred outcomes and facilitate the benchmarking and improvement of enhanced recovery pathways.

## Background

The rising prevalence of osteoarthritis, in Australia and other developed countries, has seen a corresponding rise in primary total hip arthroplasty (THA) and primary total knee arthroplasty (TKA) over the past two decades [1]. Since its inception in 2003, the Australian National Joint Replacement Registry has reported an increase in primary THA and TKA procedures of 108.1% and 156.2% respectively [2]. The increasing burden of these elective procedures has implications for health care costs and resources [1]; therefore, efficient provision of quality patient care is a priority. As such, enhanced recovery pathways (ERP) have been applied to several surgical procedures, including THA and TKA, to improve and streamline the delivery of patient care and reduce hospital length of stay (LOS).

Initially described by Kehlet [3], ERP aim to prepare patients for surgery, reduce the negative impact of surgery, and facilitate a more rapid recovery. Every step of the surgical journey, pre-operatively to post-operatively, is examined, rationalised, optimised, and standardised, resulting in a streamlined care pathway combining evidence-based clinical features with optimal organisational efficiency [4].

Although components of THA and TKA ERP have been identified, and recommendations put forward, the level of evidence supporting each of these recommendations is variable [5]. Currently, no standardised guidelines apply to each ERP component, which would more readily facilitate implementation [6]. The lack of defined guidelines, and the numerous components that ERP contain, means that successful implementation requires multidisciplinary consensus at an organisational level [6]. Achieving consensus to enable a standardised approach for each ERP component may prove challenging in many healthcare facilities. ERP literature reviews have suggested that future research should focus on understanding which pathway components contribute to improved recovery [7] and quantifying the impact of individual variables [8]. An understanding of which variables are most associated with early post-operative functional recovery may direct attention to particular pathway components, thus further improving ERP outcomes.

A recent systematic review [9] examined the prognostic relationships between patient and surgical factors and early post-operative functional recovery assessed using validated outcome measures. The review found strong evidence that comorbidity status (determined by American Society of Anaesthesiologists, ASA grade) and pre-operative function (assessed by the Timed Up and Go test, TUG) are prognostic for inpatient functional recovery following TKA. No such evidence was found for patient-related prognostic factors for inpatient recovery following THA, and no surgical factors were found to be independently prognostic for inpatient recovery following either procedure. However, limited evidence did suggest ERP may facilitate functional recovery in the TKA population. None of the studies included in the review collected data within the last 5 years, and therefore, the potential impacts of more recent surgical advances including muscle-sparing approaches and robot-assisted surgery were not assessed.

Thus, the primary aims of this study were to examine the relationships between patient-related and surgical factors and inpatient functional recovery following THA and TKA, where functional recovery was assessed using validated functional performance measures appropriate to the early post-operative period, and, based on these findings, to develop prognostic models for inpatient functional recovery. Secondary aims were to identify patient-related, surgical, or post-operative factors associated with acute hospital LOS and to assess the relationships between functional performance measures assessed on the 2nd post-operative day (POD) and longer-term (6-month) patient-reported functional outcomes following THA and TKA.

## Methods

### Research design and setting

This prospective cohort study was conducted at The Wesley Hospital, Brisbane, an Australian privately funded, not for profit hospital. The ERP applied to this patient cohort is partially standardised, allowing for individual preferences of surgeons and anaesthetists. Ethics approval was obtained for this study from the Uniting Care Human Research Ethics Committee (HREC no. 2016.09.187) and Bond University Human Research Ethics Committee (HREC no. 15685).

### Participants

All patients undergoing elective, primary, unilateral THA and TKA between 1 May 2018 and 30 April 2019 were considered for inclusion. Potential participants were provided with information about the study for consideration prior to their attendance at pre-admission clinic, where eligibility criteria were applied and written informed consent was obtained. Patients were excluded if they were undergoing uni-compartmental, bilateral, or revision arthroplasty; not reviewed pre-operatively or unable to perform the assessments of pre-operative function; considered inappropriate to participate in the existing ERP due to multiple complex comorbidities; or identified to have significant language or cognitive barriers. A two-stage screening process was used to confirm adequate cognitive function (Appendix 1). The first stage involved verbal screening in the pre-admission clinic by an occupational therapist, and a Mini-Mental State Examination (MMSE) [10] was performed for any potential participants who reported difficulty with memory or cognition. Secondly, an MMSE was undertaken for any participants whom the treating physiotherapist observed poor recall or carry-over between treatment sessions, which appeared to be limiting post-operative progression. The exclusion criteria were devised to ensure homogeneity of participants with regard to pre-operative education and surgical procedure and to exclude patients with factors reasonably considered to influence their ability to follow usual instruction or participate in the usual post-operative physiotherapy care as part of the existing ERP. All patients received usual pre-operative and post-operative care regardless of their participation in the study, with the only difference being that data pertaining to the potential prognostic factors were extracted from the medical charts of participants.

### Prognostic factors

Patient-related factors (Table 2) and potentially modifiable peri-operative and post-operative factors (Table 3) associated with the existing ERP were selected as the potential prognostic factors to be investigated.

### Outcome measures

#### Primary outcome measure

The primary outcome measure was inpatient functional recovery assessed on POD 2 using the modified Iowa Level of Assistance Scale (mILAS) [11]. The mILAS (Appendix 2) is an easily performed 6-item functional performance measure that assesses 4 activities of daily living (ADL; supine to sitting, sit to stand, walking, and negotiation of a single step), walking distance, and required mobility aid. Each item is scored 0–6, with a maximum possible total score of 36; higher scores indicate greater functional dependence. The mILAS has demonstrated validity in assessing readiness for discharge, with a statistically significant difference in median scores of 17 points observed between patients considered ready for discharge (median score 0, IQR 0–4.25) and those deemed not yet ready for discharge (median score 17, IQR 12–23) [11]. The mILAS is responsive, with a minimal detectable change (MDC) of 5.8 points and large changes in scores typically evident over the course of an acute hospital admission; furthermore, it has excellent inter-rater reliability (intraclass correlation coefficient; ICC = 0.975) [11].

#### Secondary outcome measures

##### Timed Up and Go test, 10-metre walk test

POD 2 inpatient functional recovery was also assessed using the Timed Up and Go (TUG) test [12] and 10-metre walk test (10mWT) [13]. The TUG is a reliable test of functional mobility in patients following TKA, with excellent test-retest reliability (ICC = 0.98) and a MDC of 2.27 s [14], and has been demonstrated to predict both short-term [15, 16] and long-term function following lower limb arthroplasty [17, 18]. The 10mWT is a reliable measure of gait speed [19]. For both the TUG and 10mWT, a higher score (in seconds) indicates a slower gait speed, and each is an independent predictor of general health decline, ADL difficulty, and falls, in older community-dwelling adults [20].

##### Longer-term functional outcomes

Longer-term functional outcomes were assessed using patient-reported outcome measures (PROM), including the Oxford Hip Score (OHS) [21] or Oxford Knee Score (OKS) [22], and the EuroQol-5 Dimension visual analogue scale (EQ-5D VAS) [23], each administered by telephone. The OHS and OKS are joint-specific PROM designed to assess pain behaviour and ability to perform ADL following THA and TKA, with higher scores indicating greater function [24]. The OHS and OKS have undergone extensive reliability and validity testing [24] and have been used in multiple studies, to benchmark arthroplasty outcomes in the UK and Australian National Joint Replacement Registries. The minimal important changes (MIC) for assessment at the group level are 11 and 9 points for the OHS and OKS, respectively [25]. For assessment of individual patients, the MIC are 8 and 7 points for the OHS and OKS, respectively [25]. The distribution-based minimal detectable change (MDC90) estimates were 5 and 4 points, for the OHS and OKS, respectively [25]. The English language versions, adapted for use in Australia, were used in this study, and scoring was undertaken per the respective user guides [26, 27].

The EQ-5D-5L is a widely used PROM designed to provide a simple, generic measure of health [23]. The VAS component comprises a 20-cm vertical scale numbered from 0 to 100, 0 indicating “the worst health you can imagine” and 100 “the best health you can imagine”. Participants scoring less than 100 were asked to identify the aspect of their health responsible for generating the response. This was in order to distinguish whether the site of arthroplasty (or another aspect of general health) was the primary factor impacting their score on the EQ-5D VAS. It has previously been demonstrated that TKA functional outcomes measured using the OKS at 12 months post-operatively [28] were influenced by post-operative general physical health. As such, the EQ-5D VAS was used to assess general health as a potential contributor to functional outcomes of the participants.

##### Length of stay

Length of stay (LOS) was calculated as the number of nights spent in the acute hospital setting. Despite previous studies indicating that LOS is influenced by many factors other than the physical function of the patient [29–34], LOS remains a commonly used outcome measure for evaluating the success of ERP and benchmarking performance amongst healthcare organisations and thus was recorded for completeness.

### Procedure

All participants underwent primary, unilateral TKA or THA procedures and received usual pre- and post-operative care in The Wesley Hospital, consistent with the existing ERP, under the direction of their treating surgeon and independent of the research. Usual physiotherapy care involved day of surgery (DOS) mobilisation (as appropriate), bidaily physiotherapy on POD 1–3 (including weekends), and daily physiotherapy on subsequent days (at the discretion of the treating physiotherapist) until time of discharge or transfer to inpatient rehabilitation. Physiotherapy incorporated range of motion and strengthening exercises, transfer practice, gait re-education, progression of mobility aids and distances walked, stairs practice, and discharge planning.

Assessments of function were undertaken at pre-determined time points (Table 1). Pre-operative function was assessed 1–4 weeks pre-operatively during a usual pre-admission appointment. Assessments of post-operative function were conducted during usual post-operative physiotherapy care, on the afternoon of POD 2, and on the morning of discharge from the orthopaedic ward. The TUG and 10mWT were assessed at each time point only if the participant was judged to be able to perform the test safely and independently (using their customary mobility aid). For both the TUG and the 10mWT, time was recorded with a stopwatch (in seconds), and participants were instructed to perform the tests as quickly as possible, without compromising their safety. At each time point, two TUG trials were completed, and the faster of the two times was recorded. For the 10mWT, only one trial was assessed at each time point. For the mILAS, participants were scored based on the same mobility aid they used when performing the TUG and 10mWT.

**Table 1:** Time points for assessment of pain and functional measures

Time point	Assessments of pain and function
**Pre-admission clinic**	EuroQol-5 Dimension Visual Analogue Scale
	Oxford Hip or Knee Score
	Visual analogue scale
	Modified Iowa Level of Assistance Scale
	Timed Up and Go
	10-metre walk test
**Post-operative day 2**	Visual analogue scale
	Modified Iowa Level of Assistance Scale
	Timed Up and Go
	10-metre walk test
**Day of discharge**	Visual analogue scale
	Modified Iowa Level of Assistance Scale
	Timed Up and Go
	10-metre walk test
**Six months post-operative** (50% of cohort only)	EuroQol-5 Dimension Visual Analogue Scale
	Oxford Hip or Knee Score

A data collection form was devised to record the potential prognostic factors and the results of outcome measures for each participant. Information was entered into a secure database, and subsequently, all participants were de-identified prior to data analysis. Patient factors were recorded during a pre-operative subjective assessment; surgical and post-operative factors were extracted from the patient medical chart. Discharge date was recorded and defined as the date each participant was discharged from the acute orthopaedic ward to a suitable home environment or to inpatient rehabilitation. Patient readiness to discharge home was mutually determined by the patient and treating surgeon, with guidance from the treating physiotherapist based on ERP discharge criteria (Appendix 3) and independent of the research. Admission to inpatient rehabilitation was based on consideration of patients’ post-operative medical or functional status and availability of appropriate social support, and independent of the research.

Data collection and assessment of all outcome measures were conducted by qualified physiotherapy staff. If participants were unable to perform any of the functional assessments at a particular time point (Table 1), the reason for this was recorded. Longer-term functional outcomes were assessed via telephone interview at 6 months post-operatively, for participants who comprised the first half of the study cohort.

### Statistical analysis

A recruitment target of 350 participants was planned for the study, based on power calculations conducted using G*Power software (version 3.1.9.2, 2014). This number of participants allowed sufficient numbers to ensure statistical power of at least 80% to detect small to moderate levels of association between the prognostic factors of interest and the primary outcome measure, if such existed in the underlying population, using multiple linear regression analyses and a significance level of 0.05.

All statistical analyses were conducted using SPSS (IBM, version 26, 2019). Descriptive analyses were first conducted to describe the study cohort and variables of interest and to identify missing values. Distributions of all continuous prognostic factors and outcome measures were assessed, with normality and outliers assessed via visual inspection of histograms, box plots, and normal QQ plots, to inform decisions regarding the removal of outliers and approaches to statistical analysis. Independent samples *t* tests, Mann-Whitney *U* tests, and chi-square tests were used, as appropriate based on variable types and distributions, to assess baseline differences between the two surgical groups (TKA and THA) in demographics, other prognostic factors, and outcome measures.

To enable assessment of the linearity or other form of relationships between continuous prognostic and outcome variables and so inform decision-making about whether linear regression analyses would be appropriate to use to assess prognostic relationships, simple error bar charts were developed and visually inspected. For this purpose, continuous prognostic factors were first categorised into equal intervals, and then, where needed to ensure at least 10 participants in each category, two or more categories at either or both ends of the range of values for each variable were collapsed to form single categories.

Where linearity of relationships was evident, correlations between each of the ordinal or continuous prognostic factors and POD 2 mILAS were determined using Pearson’s and Spearman’s correlation analyses, as appropriate. Relationships between each of the nominal (dichotomous) prognostic factors and POD 2 mILAS were assessed using point biserial correlation analyses. Only prognostic factors that were significantly associated with POD 2 mILAS at the 0.1 level of statistical significance were included in subsequent multiple linear regression analyses. Nominal prognostic factors with sub-groups of less than 30 participants were also excluded from subsequent analyses due to the impacts of small sub-groups on statistical power to detect associations.

Prior to the conduct of multiple linear regression modelling, Pearson’s correlation analyses were undertaken to identify collinearity among continuous prognostic factors. Similarly, point biserial correlation analyses were undertaken to determine whether any dichotomous prognostic factors were substantially correlated with the continuous prognostic factors. Pairs of factors for which the correlation analyses yielded *r* > 0.7 were identified, and in any such instances, one of the two correlated factors was removed from the subsequent regression analyses, based on pragmatic considerations which were recorded. Backward, stepwise, multiple linear regression analysis was then used to determine the combination of prognostic factors that best predicted POD 2 mILAS, with the level of statistical significance set at 0.05 for retention of any prognostic factor in the final regression model. Regression models were determined in this way for the whole cohort and separately for each of the THA and TKA cohorts.

## Results

**Fig. 1:**
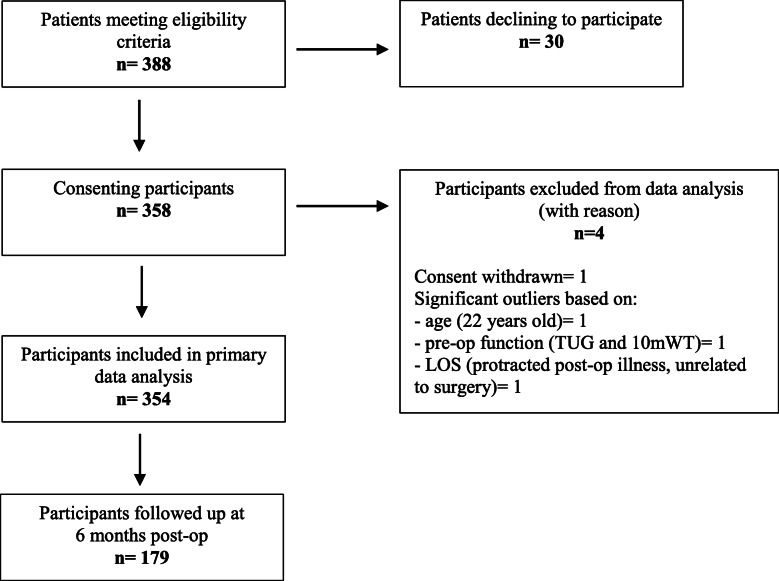
Study flow diagram

The patient and participant flow through the study is depicted in Fig. 1.

Descriptive statistics for patient characteristics assessed pre-operatively are presented in Table 2. Statistically significant differences existed between THA and TKA groups for mean body mass index (BMI) (*p* = 0.002), ASA grade distribution (*p* < 0.001), patient-reported pre-operative function as determined by mean OHS or OKS scores (*p* = 0.02), and mean Risk And Prediction Tool (RAPT) score (*p* = 0.03). However, with the exception of ASA grade distributions, these differences were not of a sufficient magnitude to be considered clinically important.

**Table 2:** Descriptive statistics. Patient characteristics, assessed pre-operatively

Patient characteristic	Total cohort, *N* = 354	TKA, *n* = 238 (67.2%)	THA, *n* = 116 (32.8%)	*p* value
**Age** (years), mean (SD)	68.9 (9.2)	69.5 (8.9)	67.7 (9.9)	0.09
**Sex**, *N* (%)				0.30
Female	185(52.0%)	129 (54.2%)	56 (48.3%)	
Male	169 (48.0%)	109 (45.8%)	60 (51.7%)	
**BMI** (kg/m^2^)				
Median (IQR)	30.0 (26.6, 33.6)	30.3 (27.0, 34.5)	28.4 (26.1, 31.3)	0.002*
Range	(18.1–48.8) Missing: *n* = 1 (0.3%)	(18.1–48.8)	(18.9–45.8)	
**ASA grade,** *N* (%)				
1	27 (8.2%)	12 (5.5%)	15 (13.5%)	< 0.001*
2	180 (54.4%)	111 (50.5%)	69 (62.2%)	
3	122 (36.9%)	97 (44.1%)	25 (22.5%)	
4	2 (0.6%)	0 (0%)	2 (1.8%)	
Median (IQR)	2 (2, 3) Missing: *n* = 23 (6.5%)	2 (2, 3)	2 (2, 2)	
**Pre-operative Hb** (g/L)				
Mean (SD)	137.9 (11.8) Missing: *n* = 4 (1.1%)	137.5 (11.6)	138.7 (12.1)	0.40
**Pre-operative pain** (VAS 0–100)				0.05
Mean (SD)	38.9 (26.3) Missing: *n* = 6 (1.7%)	37.0 (26.7)	42.8 (25.0)	
**Pre-operative function**				
**OKS/OHS** (0–48)				
Mean (SD)	25.5 (8.0) Missing: *n* = 2 (0.6%)	26.0 (8.0)	23.7 (7.9)	0.02*
**EQ-5D VAS** (0–100)				
Median (IQR)	80 (70, 90)	80 (70, 90)	80 (65, 90)	0.11
Range	(10–100) Missing: *n* = 4 (1.1%)	(10–100)	(18–100)	
**mILAS (0-36)**				
Median (IQR)	0 (0.0, 0.0)	0 (0.0, 0.0)	0 (0.0, 0.0)	0.16
Range	(0–11)	(0–11)	(0–11)	
**TUG (s)**				
Median (IQR)	8.79 (7.41, 11.09)	8.76 (7.58, 11.05)	8.98 (7.17, 11.28)	0.56
Range	(4.39–30.93)	(4.97–29.29)	(4.39–30.93)	
**10mWT (s)**				
Median (IQR)	7.61 (6.49, 9.40)	7.65 (6.52, 9.40)	7.58 (6.44, 9.42)	0.71
Range	(4.23–27.02)	(4.23–22.94)	(4.75–27.02)	
**RAPT score**				
Mean (SD)	9.48 (2.1) Missing: *n* = 5 (1.4%)	9.3 (2.2)	9.8 (2.0)	0.03*

Missing data: was omitted during pre-admission assessment or could not be extracted from patient medical chart

*ASA* American Society of Anaesthesiologists, *BMI* body mass index, *EQ-5D VAS* EuroQol-5 Dimension Visual Analogue Scale, *Hb* haemaglobin, *IQR* inter-quartile range, *mILAS* modified Iowa Level of Assistance Scale, *OKS* Oxford Knee Score, *OHS* Oxford Hip Score, *RAPT* Risk Assessment and Prediction Tool, *THA* total hip arthroplasty, *TKA* total knee arthroplasty, *TUG* Timed Up and Go test, *VAS* visual analogue scale, *10mWT* ten-metre walk test

*Statistical significance assessed at the 0.05 level. *p* values were derived from an independent-samples *t*-test, Mann-Whitney *U* test, or chi-square test comparing TKA and THA cohorts, as appropriate for variable type

Descriptive statistics for surgical prognostic factors extracted from medical records are presented in Table 3. A statistically significant difference was identified between THA and TKA groups only for anaesthetic method–general anaesthetic (GA) only (*p* = 0.04).

**Table 3:** Descriptive statistics. Surgical prognostic factors

Surgical factor	Total cohort, *N* = 354	TKA, *n* = 238 (67.2%)	THA, *n* = 116 (32.8%)	*p* value
**Tourniquet duration**				N/A
(mean [SD] min)		59.0 (21.5) *n* = 231 (missing *n* = 7)		
**Tourniquet pressure**				N/A
(mean [SD] mmHg)		292.5 (29.1) *n* = 231 (missing *n* = 3)		
**Surgical approach**, *N* (%)				N/A
Parapatellar TKA		228 (95.8%)		
Subvastus TKA		10 (4.2%)		
Posterior approach THA			71 (61.2%)	
Direct anterior approach THA			22 (19.0%)	
Direct superior approach THA			23 (19.8%)	
**Robot-assisted surgery**, *N* (%)				0.16
No	318 (89.8%)	210 (88.2%)	108 (93.1%)	
Yes	36 (10.2%)	28 (11.8%)	8 (6.9%)	
**Duration of surgery** (min)				0.67
Median (IQR)	79 (70, 93)	79 (71, 91)	79 (67, 100)	
Range	(37–178)	(37–178)	(49–167)	
**Anaesthetic method**, *N* (%)				
GA only	97 (27.4%)	57 (23.9%)	40 (34.5%)	0.04*
GA + spinal LA	198 (55.9%)	145 (60.9%)	53 (45.7%)	0.07
Sedation + spinal LA	55 (15.5%)	34 (14.3%)	21 (18.1%)	0.35
Other (intrathecal morphine)	4 (1.1%)	2 (0.8%)	2 (1.7%)	N/A
**Initial analgesia**, *N* (%)				
Intra-operative LIA	294 (83.1%)	214 (89.9%)	80 (69.0%)	< 0.001*
PCA	76 (21.5%)	49 (20.6%)	27 (23.3%)	0.56
Oral analgesia only	27 (7.6%)	7 (2.9%)	20 (17.2%)	< 0.001*
Intra-articular catheter		113 (47.5%)	N/A	
Single-shot regional block		31 (13.0%)	N/A	
Ambulatory regional block		25 (10.5%)	N/A	

Missing data: could not be extracted from patient medical chart

*GA* general anaesthesia, *IQR* inter-quartile range, *LA* local anaesthesia, *LIA* local infiltration analgesia, *PCA* patient-controlled analgesia, *THA* total hip arthroplasty, *TKA* total knee arthroplasty

*Statistical significance assessed at the 0.05 level. *p* values were derived from an independent-samples *t* test, Mann-Whitney *U* test, or chi-square test comparing TKA and THA cohorts, as appropriate for variable type

### Primary outcome measure

#### POD 2 mILAS

For the overall cohort, POD 2 mILAS scores ranged from 0 to 27, with a mean score (± SD) of 11.34 (± 6.2) points, and there were no missing values. The mean score for the THA group was 9.85 (± 6.0) and for the TKA group 12.06 (± 6.1), giving a statistically significant mean difference between the 2 groups of 2.21 points (95% CI, 0.85, 3.57), *t*(352) = 3.187, *p* = 0.02. Linearity was established for the relationships between POD 2 mILAS and all continuous and ordinal prognostic factors. However, following visual inspection of boxplots, three significant outliers were identified, and these participants were removed from further analysis with reasons recorded (Fig. 1). The levels of association between the individual prognostic factors and POD2 mILAS scores are presented in Table 4.

**Table 4:** Correlation between individual potential prognostic factors and POD 2 mILAS scores

Potential prognostic factors	*r* or *r*_*S*_	*p* value
Patient-related factors		
Age	0.34	< 0.001*
Pre-operative Hb	− 0.19	< 0.001*
RAPT score	− 0.39	< 0.001*
Pre-operative patient-reported function (OKS/OHS)	− 0.16	< 0.001*
Pre-operative function (mILAS)	0.20	< 0.001*
Pre-operative function (TUG)	0.33	< 0.001*
Pre-operative function (10mWT)	0.34	< 0.001*
Gender (0 = female, 1 = male)	− 0.19	< 0.001*
ASA	0.17	0.002*
Pre-operative patient-reported general health (EQ-5D VAS)	− 0.11	0.04*
Pre-operative pain (VAS)	0.09	0.11
BMI	0.05	0.34
Surgical factors		
Surgical approach (THA cohort only) (0 = PA, 1 = DAA or DSA)	− 0.37	< 0.001*
LIA use	− 0.21	< 0.001*
Surgery duration	− 0.24	< 0.001*
PCA use	0.18	0.001*
Arthroplasty site (0 = THA, 1 = TKA)	0.17	0.002*
Intra-articular catheter (TKA cohort only)	− 0.19	0.003*
GA only	− 0.13	0.02*
Sedation and spinal anaesthesia	0.09	0.09*
Robot-assisted surgery	− 0.09	0.09*
Single-shot regional block (TKA cohort only)	0.08	0.21
Oral analgesia only	0.06	0.25
GA and spinal anaesthesia	0.03	0.53
Surgical approach (TKA cohort only) (1 = parapatellar approach, 2 = subvastus approach)	− 0.04	0.54
Ambulatory regional block (TKA cohort only)	0.04	0.57
Tourniquet pressure (TKA cohort only)	− 0.02	0.76
Tourniquet duration (TKA cohort only)	− 0.02	0.80

*ASA* American Society of Anaesthesiologists, *BMI* body mass index, *DAA* direct anterior approach, *DOS* day of surgery, *DSA* direct superior approach, *EQ-5D VAS* EuroQol-5 Dimension Visual Analogue Scale, *Hb* haemaglobin, *GA* general anaesthesia, *LIA* local infiltration analgesia, *mILAS* modified Iowa Level of Assistance Scale, *OKS* Oxford Knee Score, *OHS* Oxford Hip Score, *PA* posterior approach, *PCA* patient-controlled analgesia, *POD* post-operative day, *RAPT* Risk Assessment and Prediction Tool, *THA* total hip arthroplasty, *TKA* total knee arthroplasty, *TUG* Timed Up and Go test, *VAS* visual analogue scale, *10mWT* ten-metre walk test

*Statistical significance assessed at the 0.1 level. *p* values were derived from Pearson’s correlation, Spearman’s correlation, or point biserial correlation, as appropriate for variable type

BMI, pre-operative pain (VAS), tourniquet duration, tourniquet pressure, oral analgesia only, TKA surgical approach, single-shot regional block, and regional block infusion were excluded from the subsequent regression analyses as no statistically significant association was identified between these prognostic factors and POD 2 mILAS scores at the 0.1 level of significance. ASA grade was not included in the regression model due to too few case numbers in ASA grades 1 and 4. Instead, Spearman’s correlation analysis was used to gauge the strength of the relationship between ASA grade and POD 2 mILAS scores, and a weak but statistically significant positive correlation (*r*_*S*_ = 0.17 (329), *p* = 0.002) was identified. Robot-assisted surgery was removed from TKA and THA analyses due to too few participants having undergone this type of surgery, once the cohort was split by surgical type. Pre-operative TUG time and pre-operative 10mWT time were highly correlated, *r* = 0.880, and so, the prognostic factor pre-operative 10mWT was not included in the subsequent regression analyses. This decision to remove the 10mWT rather than TUG from the subsequent regression analyses was made due to variation within the literature regarding the methodology of the 10mWT [35], and thus, its implementation in clinical practice was considered to be potentially less standardised than implementation of the TUG.

The final regression model for prediction of POD 2 mILAS scores in the overall combined TKA and THA cohort, based on significant prognostic factors, is depicted in Table 5, with *R*^2^ of 34.7% and adjusted *R*^2^ of 33.1%, reflecting a medium effect size [36], and with *F*(8, 329) = 21.882, *p* < 0.001. POD 2 mILAS scores (i.e. level of functional dependence of the patient) increased an average of 0.20 points for every year of age, after the other significant prognostic factors were considered (Table 5). POD 2 mILAS scores were on average 1.67 points higher for females than males and decreased an average of 0.05 points for every additional point reported on the EQ-5D VAS general health scale (Table 5). Pre-operative TUG time was a further significant prognostic factor, with POD 2 mILAS scores increasing on average 0.45 points for every additional second a patient required to complete the TUG (Table 5). Among the surgical factors, arthroplasty site was a significant prognostic factor, with POD 2 mILAS scores on average 2.21 points higher in patients who underwent a TKA rather than a THA procedure (Table 5). POD 2 mILAS scores were on average 2.07 points lower in patients who received general anaesthesia (GA) only, when compared to those who received other forms of anaesthesia (Table 5). Similarly, POD 2 mILAS scores were on average 3.02 points lower in patients who received local infiltration analgesia (LIA), when compared to those patients who did not, and POD 2 mILAS scores were on average 2.02 points higher in patients who received post-operative analgesia via patient-controlled analgesia (PCA) when compared to those patients who did not (Table 5).

**Table 5:** Final prognostic model for post-operative day 2 functional recovery (POD 2 mILAS) in overall cohort

Prognostic factors	Unstandardized regression coefficient (*B*)	Standard error of the coefficient (SE_*B*_)	95% CI for *B*	Standardized coefficient (*ß*)	*p* value
Constant/intercept	− 0.906	2.550	− 5.923, 4.111		0.723
Age	0.201	0.032	0.138, 0.264	0.297	< 0.001
Gender (0 = female, 1 = male)	− 1.671	0.571	− 2.795, − 0.547	− 0.134	0.004
Pre-operative patient-reported general health (EQ-5D VAS/100)	− 0.051	0.020	− 0.090, − 0.013	− 0.126	0.009
Pre-operative function (TUG, sec)	0.453	0.094	0.269, 0.637	0.241	< 0.001
Arthroplasty site (0 = THA, 1 = TKA)	2.223	0.631	0.982, 3.464	0.167	< 0.001
Anaesthetic—use of GA only	− 2.067	0.674	− 3.392, − 0.742	− 0.148	0.002
Initial analgesia—LIA use	− 3.022	0.848	− 4.690, − 1.355	− 0.184	< 0.001
Initial analgesia—PCA use	2.021	0.765	0.516, 3.526	0.134	0.009

Statistical significance assessed at the 0.05 level

*CI* confidence interval, *EQ-5D VAS* EuroQol-5 Dimension Visual Analogue Scale, *GA* general anaesthesia, *LIA* local infiltration analgesia, *PCA* patient-controlled analgesia, *TUG* Timed Up and Go test, *VAS* visual analogue scale

The final regression model for prediction of POD 2 mILAS scores in the TKA group, based on significant prognostic factors, is depicted in Table 6, with *R*^2^ of 36.4% and adjusted *R*^2^ of 34.1%, reflecting a medium effect size [36], and with *F*(8, 220) = 15.723, *p* < 0.001. POD 2 mILAS scores increased on average 0.18 points for every year of age and were on average 1.49 points higher for females than males (Table 6). POD 2 mILAS scores decreased on average 0.09 points for every additional point reported on the EQ-5D VAS general health scale, and similarly increased an average of 0.50 points for every additional second a patient required to complete the TUG (Table 6). POD 2 mILAS scores were on average 2.07 points lower in patients who received a GA only, when compared to those who received other forms of anaesthesia (Table 6). POD 2 mILAS scores were on average 3.62 points lower in patients who received LIA, when compared to those patients who did not, and POD 2 mILAS scores were on average 2.35 points higher in patients who received post-operative analgesia via PCA when compared to those patients who did not (Table 6). POD 2 mILAS scores were also on average 0.03 points lower for every additional minute of surgical time (Table 6).

**Table 6:** Final prognostic model for post-operative day 2 functional recovery (POD 2 mILAS) for the TKA group

Prognostic factors	Unstandardized regression coefficient (*B*)	Standard error of the coefficient (SE_*B*_)	95% CI for *B*	Standardized coefficient (*ß*)	*p* value
Constant/intercept	7.837	4.187	− 0.415, 16.090		0.063
Age	0.184	0.042	0.101, 0.267	0.263	< 0.001
Gender (0 = female, 1 = male)	− 1.487	0.696	− 2.859, − 0.116	− 0.120	0.034
Pre-operative patient-reported general health (EQ-5D VAS/100)	− 0.087	0.024	− 0.134, − 0.039	− 0.207	< 0.001
Pre-operative function (TUG, sec)	0.503	0.114	0.279, 0.728	0.263	< 0.001
Surgery duration (min)	− 0.033	0.016	− 0.064, − 0.002	− 0.125	0.038
Anaesthetic method: use of GA only	− 2.066	0.858	− 3.756, − 0.375	− 0.143	0.017
Initial analgesia: LIA use	− 3.619	1.193	− 5.971, − 1.267	− 0.179	0.003
Initial analgesia: PCA use	2.345	0.959	0.455, 4.235	0.154	0.015

Statistical significance assessed at the 0.05 level

*CI* confidence interval, *EQ-5D VAS* EuroQol-5 Dimension Visual Analogue Scale, *GA* general anaesthesia, *LIA* local infiltration analgesia, *PCA* patient-controlled analgesia, *TUG* Timed Up and Go test, *VAS* visual analogue scale

The final regression model for prediction of POD 2 mILAS scores in the THA group, based on significant prognostic factors, is depicted in Table 7, with *R*^2^ of 32.4% and adjusted *R*^2^ of 30.4%, reflecting a medium effect size [36], and with *F*(3, 105) = 16.742, *p* < 0.001. POD 2 mILAS scores decreased an average of 0.94 points for every additional point scored on the RAPT, after the other significant prognostic factors were considered (Table 7). POD 2 mILAS scores increased on average 0.36 points for every additional second a patient required to complete the TUG (Table 7). THA surgical approach was a further significant prognostic factor, with POD 2 mILAS scores on average 4.67 points higher in patients who underwent THA via a posterior surgical approach when compared to other surgical approaches (direct anterior approach or direct superior approach; Table 7).

**Table 7:** Final prognostic model for post-operative day 2 functional recovery (POD 2 mILAS) for the THA group

Prognostic actors	Unstandardized regression coefficient (*B*)	Standard error of the coefficient (SE_*B*_)	95% CI for *B*	Standardized coefficient (*ß*)	*p* value
Constant/intercept	17.614	3.824	10.031, 25.196		< 0.001
RAPT score (/12)	− 0.943	0.278	− 1.493, − 0.392	− 0.314	0.001
Pre-operative function (TUG, sec)	0.357	0.160	0.039, 0.674	0.205	0.028
Surgical approach (0 = PA, 1 = DAA or DSA)	− 4.671	1.016	− 6.686, − 2.656	− 0.373	< 0.001

Statistical significance assessed at the 0.05 level

*CI* confidence interval, *DAA* direct anterior approach, *DSA* direct superior approach, *PA* posterior approach, *RAPT* Risk Assessment and Prediction Tool, *THA* total hip arthroplasty, *TUG* Timed Up and Go test

Independent samples *t* tests and chi-square tests revealed no statistically significant differences in age, ASA grade distributions, or measures of pre-operative function, between the THA surgical approach groups—posterior approach (PA) versus direct anterior approach (DAA) or direct superior approach (DSA).

Additional analyses revealed participants who mobilised on the DOS had lower mean POD 2 mILAS scores (10.43 ± 5.8) than those who first mobilised on POD 1 (13.64 ± 6.5), with a statistically significant difference of 3.21 points (95% CI, 1.81, 4.61), *t*(352) = 4.513, *p* < 0.001. In the overall cohort, 49.2% experienced barriers to post-operative progress (Table 8). Participants who experienced post-operative progress barriers had higher mean POD 2 mILAS scores (14.65 ± 5.5) than those who did not (8.13 ± 5.0), with a statistically significant difference of 6.52 points (95% CI, − 7.62, − 5.42), *t*(352) = − 11.636, *p* < 0.001.

**Table 8:** Symptoms reported in medical records as impacting post-operative progress in overall cohort

Progress barriers	*N* (%)
Pain	100 (28.2%)
Nausea	47 (13.3%)
Dizziness/low BP/pre-syncope	53 (15.0%)
Drowsiness/fatigue	22 (6.2%)
Delirium/impulsiveness	14 (4.0%)
Wound ooze	12 (3.4%)
Anxiety	12 (3.4%)
Constipation	12 (3.4%)
Quads inhibition	9 (2.5%)
Other (including systemically unwell or medical condition unrelated to surgery)	25 (7.1%)

### Secondary outcome measures

#### POD 2 TUG and POD2 10mWT

In the overall cohort, 75.7% and 76.3% of participants completed the POD 2 TUG and 10mWT, respectively. The reasons for non-completion of these outcome measures are as follows: 18.1% of the cohort failed to meet an appropriate level of functional independence, 2.5% were inadvertently omitted by the treating therapist, and approximately 3.0% were limited by symptoms including pain, nausea, dizziness, wound ooze, and diarrohea or were awaiting investigations. Due to the proportion of participants for whom this outcome data was missing, regression analyses were not completed for these outcome measures. However, Spearman’s correlation revealed a moderate correlation between POD 2 mILAS scores and scores on each of the secondary outcome measures assessed on POD 2: POD 2 TUG *r*(266) = 0.48, *p* < 0.001, and POD 2 10mWT *r*(268) = 0.39, *p* < 0.001.

#### Longer-term (6 months) PROMs

As planned, 6 months follow-up and collection of data pertaining to longer-term outcomes were completed for 179 (50.6%) of the 354 participants included in the primary analyses. For this sub-group, the changes between baseline measures and measures assessed at 6 months post-operatively are presented in Table 9. The median increases in OHS and OKS scores for the THA and TKA sub-groups, respectively, exceeded the group MICs for these measures of 11 (OHS) and 9 points (OKS) [25] (Table 9). The OHS MIC, for individuals, of 8 points [25] was exceeded by 93.3% of the THA sub-group. Similarly, 82.9% of the TKA sub-group exceeded the OKS MIC for individuals of 7 points [25] (Table 9). An increase of only 1 point in the median change score for the EQ-5D VAS (Table 9) indicated that self-reported general health status did not differ significantly between pre-operative assessment and 6 months post-operative assessment for the participants followed up at 6 months post-operatively.

**Table 9:** Differences in pre-operative and longer-term (6 months post-operative) patient-reported outcome measures

Patient-reported outcome measure	Baseline score Median (IQR) Range, *n*	6 months post-op score Median (IQR) Range, *n*	Change score^a^ Median (IQR) Range, *n*	Change score exceeded MIC At group level^b^, *n* (%) For individuals^c^, *n* (%)
**OHS**	22.0 (17.25, 29.0) 5–39, *n* = 60	46.0 (44.0, 48.0) 22–48, *n* = 61	22.5 (16.0, 28.75) 1–42, *n* = 60	60 (100%) 56 (93.3%)
**OKS**	25.0 (19.0, 32.0) 7–42, *n* = 117	43.0 (38.75, 46.0) 26-48, *n* = 118	16.0 (9.5, 23.0) − 4.0–41.0, *n* = 117	117 (100%) 97 (82.9%)
**EQ-5D VAS**	80.0 (70.0, 90.0) 25–100, *n* = 178	85.0 (75.0, 90.0) 10–100, *n* = 179	1.0 (− 5.0, 12.5) − 75–50, *n* = 178	N/A

*EQ-5D VAS* EuroQol-5 Dimension Visual Analogue Scale, *IQR* inter-quartile range, *MIC* minimal important change, *OHS* Oxford Hip Score, *OKS* Oxford Knee Score

^a^Change score is the difference between pre-operative baseline score and 6 months post-operative score

^b^For assessment at the group level, an MIC of 11 and 9 points was used for the OHS and OKS, respectively (Beard et al. [28])

^c^For assessment of individual patients, an MIC of 8 and 7 points was used for the OHS and OKS, respectively (Beard et al. [28])

A small, negative statistically significant correlation existed between POD 2 mILAS score and OKS or OHS scores at 6 months post-operatively for the overall sub-group (*r*(177) = − 0.27, *p* < 0.001), and similarly for the TKA sub-group (*r*(116) = − 0.19, *p* = 0.037). A similar small, negative correlation of borderline statistical significance was found for the THA sub-group (*r*(59) = − 0.25, *p* = 0.052). The correlation between POD 2 mILAS scores and EQ-5D VAS scores at 6 months post-operatively did not meet statistical significance for any of the studied cohorts.

For the THA sub-group, a moderate, positive, statistically significant correlation existed between OHS and EQ-5D VAS assessed at 6 months post-operatively (*r*(59) = 0.39, *p* = 0.002), and a non-statistically significant small, positive correlation was found between OKS and EQ-5D VAS assessed at the same time point for the TKA sub-group (*r*(116) = 0.16, *p* = 0.093). 44.1% of the overall sub-group reported health conditions unrelated to the arthroplasty undertaken during the study impacted their EQ-5D VAS assessed at 6 months post-operatively.

#### LOS

LOS ranged from 2 to 16 days across all participants, with a median LOS of 4 days for the overall cohort, and for each of the THA and TKA groups. Statistically significant correlations were found between multiple factors and LOS (Table 10). Patient-related factors that were significantly positively correlated with LOS included age, ASA grade, and pre-operative TUG time, 10mWT time, and mILAS score. Those significantly negatively correlated with LOS were pre-operative RAPT score, EQ-5D VAS, and OKS/OHS scores (Table 10). Surgical and post-operative factors significantly positively correlated with LOS included incidence of post-operative progress barriers and POD 2 mILAS, TUG, and 10mWT scores (Table 10). THA (rather than TKA) surgery, DAA or DSA (rather than PA) THA, use of GA alone, combined use of GA and spinal anaesthesia, intra-operative LIA use, and DOS mobilisation were all associated with shorter LOS than their alternatives (Table 10).

**Table 10:** Association between individual potential prognostic factors and length of stay in overall cohort

Potential prognostic factors	*r*_S_ or *U, Z* values IQR LOS (nights) for groups	*p* value
**Patient-related factors**		
Gender (0 = female, 1 = male)	14685.0, − 1.022 IQR female (4.0–5.0), IQR male (4.0–5.0)	0.31
Age	0.20	< 0.001**
BMI	0.06	0.29
ASA	0.12	0.03*
Hb	− 0.03	0.54
Living situation	0.04	0.51
RAPT score	− 0.27	< 0.001**
Pre-op pain (VAS)	0.08	0.15
Pre-op patient-reported general health (EQ-5D VAS)	− 0.11	0.03*
Pre-op patient-reported function (OKS/OHS)	− 0.18	0.001**
Pre-op function (mILAS)	0.20	< 0.001**
Pre-op function (TUG)	0.26	< 0.001**
Pre-op function (10mWT)	0.21	< 0.001**
**Surgical factors**		
Arthroplasty site (0 = THA, 1 = TKA)	12065.0, − 1.996 IQR THA (3.0–5.0), IQR TKA (4.0–5.0)	0.05*
Robot-assisted surgery (0 = no, 1 = yes)	5337.0, − 0.690 IQR no (4.0–5.0), IQR yes (3.25–5.0)	0.49
GA only (0 = no, 1 = yes)	10172.5, − 2.769 IQR no (4.0–5.0), IQR yes (3.0–5.0)	0.006**
GA and spinal anaesthesia (0 = no, 1 = yes)	13215.0, − 2.419 IQR no (3.0–5.0), IQR yes (4.0–5.0)	0.02*
Sedation and spinal anaesthesia (0 = no, 1 = yes)	7972.0, − 0.373 IQR no (4.0–5.0), IQR yes (4.0–5.0)	0.71
LIA use (0 = no, 1 = yes)	7150.0, − 2.398 IQR no (4.0–5.75), IQR yes (4.0–5.0)	0.02*
PCA use (0 = no, 1 = yes)	9709.0, − 1.122 IQR no (4.0–5.0), IQR yes (4.0–6.0)	0.26
Intra-articular catheter (TKA only) (0 = no, 1 = yes)	6671.0, − 0.755 IQR no (4.0–5.0), IQR yes (4.0–5.0)	0.45
Single-shot regional block (TKA only) (0 = no, 1 = yes)	2566.5, − 1.867 IQR no (4.0–5.0), IQR yes (4.0–6.0)	0.06
Ambulatory regional block (TKA only) (0 = no, 1 = yes)	2437.0, − 0.720 IQR no (4.0–5.0), IQR yes (3.0–5.5)	0.47
Surgical approach (THA only) (0 = PA, 1 = DAA or DSA)	975.5, − 3.645 IQR PA (4.0–5.0), IQR DAA or DSA (3.0–4.0)	< 0.001**
Surgery duration	− 0.03	0.52
Tourniquet duration (TKA only)	− 0.02	0.94
Tourniquet pressure (TKA only)	− 0.02	0.50
**Post-operative factors**		
DOS mobilisation (0 = no, 1 = yes)	10,659.0−2.443 IQR no (4.0–6.0), IQR yes (4.0–5.0)	0.02*
Incidence of post-operative progress barriers (0 = no, 1 = yes)	8482.5, − 7.735 IQR no (3.0–4.0), IQR yes (4.0–6.0)	< 0.001**
POD 2 mILAS	0.54	< 0.001**
POD 2 TUG	0.31	< 0.001**
POD 2 10mWT	0.28	< 0.001**

*p* values were derived from Spearman’s correlation or Mann-Whitney *U* test, as appropriate for variable type

*ASA* American Society of Anaesthesiologists, *BMI* body mass index, *DAA* direct anterior approach, *DSA* direct superior approach, *DOS* day of surgery, *EQ-5D VAS* EuroQol-5 Dimension Visual Analogue Scale, *GA* general anaesthesia, *Hb* haemaglobin, *IQR* inter-quartile range, *LIA* local infiltration analgesia, *LOS* length of stay, *mILAS* modified Iowa Level of Assistance Scale, *OKS* Oxford Knee Score, *OHS* Oxford Hip Score, *PA* posterior approach, *PCA* patient-controlled analgesia, *POD* post-operative day, *RAPT* Risk Assessment and Prediction Tool, *THA* total hip arthroplasty, *TKA* total knee arthroplasty, *TUG* Timed Up and Go test, *VAS* visual analogue scale, *10mWT* ten-metre walk test

*Statistical significance *p* < 0.05 level

**Statistical significance *p* < 0.01 level

## Discussion

This study assessed the strengths of the prognostic relationships between patient-related and surgical variables and inpatient functional recovery (as assessed by POD 2 mILAS score) and yielded prognostic models for inpatient functional recovery following THA and TKA. In addition, patient-related, surgical, and post-operative factors associated with hospital LOS were identified, and the relationships between inpatient functional outcomes and longer-term (6-month) patient-reported functional outcomes were assessed. Overall, the findings indicate that a range of patient-related factors assessed pre-operatively as well as surgical and post-operative factors were associated with inpatient functional outcomes and with LOS following THA and TKA. In addition, longer-term functional outcomes for these patients reflected their inpatient functional outcomes. These findings address a gap in the existing evidence base, highlight the importance of assessing and optimising functional outcomes in the inpatient period, and may usefully inform the further development of ERP employed for THA and TKA.

The final prognostic models for both the overall cohort and TKA group explained approximately one third of the variance in inpatient functional recovery. Both models included as significant prognostic factors patient age, sex, pre-operative patient-reported general health, pre-operative function (TUG), and GA, LIA, and PCA use. In addition to these factors, arthroplasty site was a prognostic factor for the overall cohort, and surgery duration was prognostic for the TKA group. The final prognostic model for the THA group differed, possibly due in part to the smaller number of THA participants in the overall cohort, and included pre-operative function (TUG time), RAPT score, and surgical approach. Again, the prognostic model developed for the THA group explained approximately one third of the variance in inpatient functional recovery. Importantly, noted in each of the models was the contribution of both patient-related and surgical factors. This is the first study, to our knowledge, to identify independent, potentially modifiable surgical factors prognostic for early functional recovery following TKA or THA. The recent systematic review conducted by Hewlett-Smith et al. [9] did not find evidence that any individual surgical factors (other than site of arthroplasty) were prognostic for early functional recovery following THA or TKA.

In the prognostic models for both the overall cohort and TKA group, LIA use was associated with greater functional recovery and had the greatest prognostic value in both of these models, whereas PCA use had a negative impact on predicted POD2 mILAS scores. LIA is thought to provide effective early post-operative analgesia (without motor blockade), with less incidence of post-operative complications such as nausea, and a lower requirement for supplemental oral opioids; however, there is only low-level evidence for these effects in THA [37] and TKA [38]. In addition, optimal volume, composition, and site of administration of LIA have not been confirmed [5]. Limiting use of PCA pumps in the routine arthroplasty population is strongly recommended due to associated functional impedance [5]. Although attachments were routinely removed by POD 2 in the current study, PCA use was significantly correlated with post-operative nausea (*p* < 0.001) and dizziness (*p* = 0.018) in the overall cohort, which may explain its association with reduced functional recovery.

Less expected was the association between GA use and greater functional recovery in the overall cohort and TKA group. ERP literature has traditionally supported the use of spinal anaesthesia [4, 39, 40], although a meta-analysis of 29 studies including 10,488 patients reported no significant difference in the incidence of peri-operative complications following THA and TKA when comparing neuraxial and general anaesthesia [41]. However, neuraxial methods were employed in significantly fewer patients than GA, and notably, epidural rather than spinal anaesthesia was the primary mode of neuraxial anaesthesia used [41]. Recently published anaesthesia consensus guidelines have reported low evidence for primary TKA and low to moderate evidence for primary THA in favour of neuraxial anaesthesia versus GA [42]. However, lack of detailed information regarding the potentially wide variability in GA technique, in addition to the significant evolution in GA, and the potential influence of modern GA technique on outcomes were also acknowledged [42].

Surprisingly, longer surgery duration was prognostic of greater recovery for the TKA group. It was, however, found to have the least prognostic value in this model. As surgery duration may be influenced by multiple factors including surgeon experience, anaesthetic technique, surgical approach, surgical technique, and complexity of surgical procedure, the clinical relevance of this particular finding is unclear.

In contrast, surgical approach was strongly prognostic of greater inpatient functional recovery in the THA group, making a difference of 4.7 points on the 36-point mILAS scale. This may be due to the muscle-sparing nature of the DAA and DSA compared to the PA THA, for which inpatient functional recovery was poorer. A systematic review [43] confirms that few studies have compared THA surgical approaches using inpatient function as an outcome. Achievement of early post-operative functional goals has been reported in favour of DAA compared to PA THA [16, 44, 45]. Recent guidelines, however, found inconclusive evidence regarding the effect of different surgical approaches on time to meet discharge criteria following THA in an enhanced recovery setting; adequately powered randomised controlled trials were recommended [5, 8]. Presently, this study valuably adds to the evidence available to inform practice in this area. Overall, with regard to surgical factors, our results indicate that DAA or DSA for THA, use of GA only, and LIA use were all associated with greater levels of post-operative functional recovery on POD 2, and thus warrant consideration as key ERP components.

With respect to site of arthroplasty, a statistically significant difference in POD2 mILAS scores was found in favour of the THA group; however, this may be, in part, due to the significant difference identified in ASA grade distributions between the TKA and THA groups. Although the THA group had lower pre-operative comorbidity overall (Table 1), both arthroplasty groups had a median ASA grade of 2. Additionally, the THA group had a greater proportion of patients receiving GA only (also a significant prognostic factor for POD 2 mILAS scores); however, site of arthroplasty and use of GA only were both significant independent prognostic factors within the final prognostic model for the overall cohort. Few studies have investigated site of arthroplasty as a prognostic factor for early post-operative functional outcomes. Although methodological quality limited the generalisability of the results, significantly slower functional recovery was observed in TKA patients when compared to THA patients at 1 week post-operatively [46, 47].

Interestingly, several potentially modifiable patient-related factors were significantly prognostic for POD 2 mILAS scores. These include pre-operative TUG time, RAPT score, and self-reported general health status (EQ-5D VAS). TUG time was the only prognostic factor to feature in each of the final models. Findings for the overall cohort indicate that every 5-s increase in time to complete the pre-operative TUG test equates to an increase of 2.25 points in the mean predicted POD 2 mILAS score, representing poorer functional recovery. This finding is relevant particularly for patients of lower pre-operative functional status and supports the available evidence for pre-operative conditioning. The prognostic value of pre-operative TUG time, specifically, has been supported by a strong level of evidence in TKA studies, but only limited evidence in studies of THA [9]. A further study, not included in the systematic review [9], also reported pre-operative TUG time in their final predictive model for functional recovery following THA [16].

Higher RAPT scores were strongly prognostic of greater POD 2 functional recovery in the THA model, such that a difference of 5 points on the RAPT would be associated with a mILAS difference of 4.7 points (equal to that associated with a change in THA surgical approach). Developed as a screening tool to predict discharge destination following THA and TKA, the RAPT score assigns values to the patient’s age, sex, pre-operative exercise tolerance, the necessity for a mobility aid or community services, and social support upon discharge [48]. Although the RAPT score is primarily determined by non-modifiable factors, scoring of the exercise tolerance item and potentially the mobility aid item may be improved by optimising pre-operative function. The RAPT has only been identified in one other study as a potential predictor for early functional recovery [16] and was not included in their final prediction model.

Pre-operative patient-reported general health status had limited prognostic value in the models for both the overall cohort and TKA group. As expected, poorer pre-operative general health was associated with lower levels of post-operative functional recovery; however, a difference of 20 points on the EQ-5D VAS would be required to effect a change of 1.0 and 1.8 points in mILAS score for the overall cohort and TKA group, respectively. Furthermore, only a weak, statistically significant positive correlation was identified between ASA grade and POD 2 mILAS scores. This finding is in contrast with the strong and moderate levels of evidence for TKA and for THA, respectively, previously reported for an association between comorbidity status (ASA grade) and early post-operative functional outcomes [9].

Slower functional recovery was apparent for patients of older age and female sex in the overall cohort and TKA group. To date, two systematic reviews [9, 49] have reported conflicting evidence regarding an association between age and early post-operative functional recovery following THA [9, 49] and TKA [9]. Similarly, conflicting evidence was found for sex [9, 49] in studies of THA, whilst limited evidence supported an association between female sex and reduced early post-operative functional recovery in TKA studies [9].

Taken together, the identification of these patient-related prognostic factors supports pre-operative screening to identify patients of older age, female sex, poorer pre-operative health and functional status, and scoring lower on the RAPT as they may be less likely to achieve an accelerated recovery per many ERP. The use of these prognostic models during pre-operative screening may facilitate organisational efficiency by assisting in the prediction of patient flow. Telehealth may be a viable option for pre-operative screening in patients with reduced access to services [50] and avoids a significant cost burden to both patients and healthcare organisations [51]. Prehabilitation may significantly impact pre-operative TUG time, as well as exercise tolerance, thus potentially improving RAPT score and general well-being. As per the prognostic model, improvement in pre-operative function would result in changes to predicted early post-operative functional recovery.

Few studies, to our knowledge, have linked inpatient post-operative function to longer-term outcomes. Our results are similar to Bade et al. [18] who found acute functional performance to be predictive of functional performance at 6 months post-operatively following TKA, although pre-operative functional performance was found to be a stronger predictor. The small association between POD 2 mILAS scores and patient-reported functional outcomes assessed via OKS and OHS at 6 months post-operatively found for the overall cohort and the TKA sub-group warrants further research with larger cohorts. Inpatient function does not appear to be associated with longer-term general health. However, the EQ-5D VAS requires the respondent to rate their health “today”, thus providing a very specific “snap-shot” of health status, which may be influenced by numerous factors. In addition, although a positive correlation existed between 6 months post-operative OHS and OKS scores and EQ-5D VAS assessed at the same time, 44.1% of the overall sub-group reported health conditions other than the arthroplasty undertaken during the study impacted their EQ-5D VAS. Moreover, general health ratings remained largely static pre-operatively to post-operatively despite large improvements in joint-specific functional assessments (OHS and OKS).

Inpatient functional performance was significantly correlated with LOS in the overall cohort, and similar results have been reported by Poitras et al. [15]. Higher POD 2 mILAS scores may be a useful clinical indicator of patients at risk of prolonged LOS, enabling prompt post-operative planning for patients who are recovering function more slowly than expected. Weak correlations between all patient-related factors and LOS were identified in this study. This is in contrast to previously reported strong level evidence for higher ASA grade, greater number of comorbidities, and presence of heart or lung disease as predictors of longer LOS following THA [49]. THA surgical approach was the only surgical factor in this study to be moderately correlated with LOS. PA THA was associated with longer LOS than DAA or DSA. A study of 5341 THA procedures also found patients who received DAA or DSA THA had statistically significantly shorter LOS and a higher rate of discharge directly home [52].

Strengths of this study include the spectrum of patient and surgical prognostic factors and the use of standardised, validated functional performance measures which are clinically relevant, easily integrated into routine post-operative assessment, and appropriate to the time point at which they were assessed. The mILAS [11] was selected as the primary outcome measure as it incorporates tasks reflective of the ADL necessary to safely discharge home [53]. Versions of the mILAS have been used in similar studies; however, its implementation is heterogeneous and has thus limited the comparison of results. The study cohort is believed to be reflective of the general arthroplasty population, with few exclusion criteria implemented. To control for the potential influence of patient expectation on LOS, only patients who attended pre-admission clinic and received pre-operative education were included in the study.

There are several limitations of this study. The prognostic models are based on data from a single centre which has implemented a partially standardised ERP. To increase generalisability, the models require internal and external validation preferably in a multi-centre study, similarly involving non-standardised ERP. POD 2 was chosen as the time point to assess functional recovery to enable comparisons with studies examining ERP outcomes where a LOS of 2 days has been reported. However, with THA and TKA being performed as ambulatory surgery in some organisations, further studies with earlier post-operative assessment time points are needed. While LIA use was identified as a significant prognostic factor, variations in site of administration, volume, and content of LIA administered were not accounted for in this study. Similarly, differences in dosage parameters, content, or duration of PCA use were not addressed; thus, further research is required to determine the impact of these variables. The impact of robotic-assisted surgery could not be fully assessed due to its application in only a small volume of the study cohort. Currently, in this facility, robot-assisted surgery is primarily used for uni-compartmental knee arthroplasty which, to maximise homogeneity, was not included in this cohort. Due to insufficient case numbers, the role of muscle-sparing approaches for TKA was also not able to be assessed; thus, these surgical factors warrant further research. Further research is also necessary to determine cut-off points for age, pre-operative TUG time, pre-operative EQ-5D VAS, and RAPT score to further guide pre-operative screening.

Due to time constraints, only the first half of the study cohort was assessed for longer-term functional outcomes. Due to the natural evolution of enhanced recovery techniques during the period of data collection, this sample may have varied slightly, with regard to surgical factors, from the remainder of the cohort. To reduce the potential for missing data, participants were not required to attend the hospital for assessment of longer-term functional outcomes. Therefore, inpatient function and longer-term functional outcomes could not be directly compared due to the variance in outcome measures assessed. However, inpatient function and longer-term functional outcomes could both be directly compared to pre-operative functional outcomes.

Validated tools for assessing short-term post-operative function following lower limb arthroplasty are lacking [11, 54]. In the absence of a gold standard for evaluating functional recovery in acute hospital inpatients, the mILAS [11] was used in this study. However, a 30-point version of the mILAS has recently been described and validated by Elings et al. [55] which may be of greater clinical relevance as it only assesses functional tasks. Implementation of a valid, standardised performance measure, such as this newer version of the mILAS, would assist in objective assessment of post-operative functional recovery, identification of patients at risk of prolonged LOS, and evaluation of ERP interventions [54], and also facilitate the benchmarking of patient-centred outcomes between organisations.

## Conclusions

This study identified several patient-related and surgical factors prognostic for early post-operative functional recovery. Patient-related factors included in the final prognostic models for the overall cohort and TKA group were age, sex, pre-operative general health status, and pre-operative TUG time. Pre-operative TUG time and RAPT score were prognostic in the final model for the THA group. Surgical prognostic factors for the overall cohort and TKA group were use of GA only, LIA use and PCA use, with the addition of arthroplasty site in the model for the overall cohort, and surgery duration in the TKA group. Surgical approach was the only surgical prognostic factor in the model for the THA group. THA surgery was prognostic for greater functional recovery at POD 2 than TKA surgery. Several patient-related, surgical, and post-operative factors were associated with acute hospital LOS. A correlation was found between functional ability at POD 2 and OKS/OHS, assessed at 6 months post-operatively. Validation of these findings is required, and assessment time points earlier in the post-operative period could be implemented. Prognostic models may facilitate the prediction of inpatient flow thus optimising organisational efficiency. In addition, surgical prognostic factors warrant consideration as potentially key ERP elements, associated with early functional recovery. Standardised functional outcome measures are needed to evaluate patient-centred ERP outcomes and to facilitate the processes of benchmarking, auditing, and improving ERP.

## Data Availability

Access to the dataset generated and analysed during the current study, subject to necessary ethics approvals, can be obtained by contacting the corresponding author (NHS). This dataset is not publicly available due to the presence of information that could compromise research participant privacy/consent.

## Appendix 1: Two-stage process for exclusion of patients with cognitive impairment

### 1st stage: Pre-admission screening

Patients were screened by an occupational therapist (OT) during their pre-admission interview. Initial cognitive screening occurred via the OT’s observation of the patient during the interview as well as the following screening question. “Do you have any difficulties with your thinking or memory?”

If a *Yes* response was elicited with the above cognitive screening question:

The patient was flagged by the OT, and the Mini-Mental State Examination (MMSE) [10] was implemented by the patient’s treating physiotherapist on the acute orthopaedic ward during hospital admission. Patients scoring 23 or less, out of a maximum possible score of 30 (indicating cognitive impairment), were subsequently excluded from the research study, and on that basis, their data was not included in the data set or analysis.

If a *No* response was elicited with the above cognitive screening question:

The patient was considered eligible to consent to participate in the research study. However, the OT flagged any patients whom exhibited signs of cognitive impairment during the interview despite answering “No” to the screening question, and the MMSE was performed by the patient’s treating physiotherapist on the acute orthopaedic ward during hospital admission. Patients scoring 23 or less were subsequently excluded from the research study, and on that basis, their data was not included in the data set or analysis.

### 2nd stage: Noted poor post-operative progression

Any research study participant identified by their treating physiotherapist as not following a usual post-operative recovery due to possible cognitive impairment undertook an MMSE on the acute orthopaedic ward. Patients scoring 23 or less were subsequently excluded from the research study, and on that basis, their data was not included in the data set or analysis.

## Appendix 2

**Table 11:** Modified Iowa Level of Assistance Scale (Kimmel et al. [12])

Score	Amount of assistance	Items 1–4	Item 5	Item 6
**0**	Independent	No assistance or supervision is necessary to safely perform the activity (with or without an assistive device/aid)	> 40 m	No assistive device
**1**	Standby	Nearby supervision is required; no contact is necessary	26–40 m	1 stick or crutch
**2**	Minimal	One point of contact is necessary, including helping with the application of the assistive device, getting legs on/off leg rest, and stabilising the assistive device	10–25 m	2 sticks
**3**	Moderate	Two points of contact needed (1–2 people)	5–9 m	2 elbow crutches
**4**	Maximal	Significant support—3 or more points of contact (> 1 person)	3–4 m	2 axillary crutches
**5**	Failed	Attempted activity but failed with maximal assistance	2 m	Frame (standard or wheelie)
**6**	Not tested	Test was not attempted due to medical reasons or reasons of safety	< 2 m	Gutter/platform frame, standing lifter, hoist, or unsafe to use aid

Modified Iowa Level of Assistance Scale items: 1—supine to sitting on the edge of the bed, 2—sit to stand, 3—walking, 4—negotiation of one step, 5—walking distance, 6—assistive device used

## Appendix 3

**Table 12:** The Wesley Hospital Enhanced Recovery Pathway (ERP) discharge criteria for THA and TKA

Allied health	Independent with transfers in/out of bed and chair
	Walking independently with aid
	Able to negotiate stairs (with supervision, if needed)
	Equipment and follow-up physiotherapy organised
**Nursing**	Showering
	Toileting (bowels opened)
	Pain adequately controlled
	Wound and dressings reviewed (as appropriate)
	Discharge Planning (e.g. services organised if applicable, support person contacted)
**Patient**	Accepting of discharge plan

Version 1.0 12/2015

## Abbreviations

ADL: Activities of daily living
ASA: American Society of Anaesthesiologists
BMI: Body mass index
DAA: Direct anterior approach
DSA: Direct superior approach
DOS: Day of surgery
EQ-5D VAS: EuroQol 5 Dimension Visual Analogue Scale
ERP: Enhanced Recovery Pathway
GA: General anaesthesia
Hb: Haemaglobin
IQR: Inter-quartile range
LIA: Local infiltration analgesia
LOS: Length of stay
MDC: Minimal detectable change
MIC: Minimal important change
mILAS: Modified Iowa Level of Assistance Scale
MMSE: Mini-Mental State Examination
OHS: Oxford Hip Score
OKS: Oxford Knee Score
OT: Occupational therapist
PA: Posterior approach
PCA: Patient-controlled analgesia
POD: Post-operative day
PROM: Patient-reported outcome measure
THA: Total hip arthroplasty
TKA: Total knee arthroplasty
TUG: Timed Up and Go
VAS: Visual analogue scale
10mWT: Ten-metre walk test
